# Crystal structure of the RNA-recognition motif of *Drosophila melanogaster* tRNA (uracil-5-)-methyltransferase homolog A

**DOI:** 10.1107/S2053230X24000645

**Published:** 2024-01-25

**Authors:** Monika Witzenberger, Robert Janowski, Dierk Niessing

**Affiliations:** a Institute of Structural Biology, Molecular Targets and Therapeutics Center, Helmholtz Zentrum München, Ingolstaedter Landstrasse 1, 85764 Munich, Germany; b Institute of Pharmaceutical Biotechnology, Ulm University, James-Franck-Ring N27, 89081 Ulm, Germany; Sungkyunkwan University School of Medicine, Republic of Korea

**Keywords:** RRMs, TRMT2A, methyltransferases, X-ray crystallography, *Drosophila melanogaster*, neuro­degenerative disease

## Abstract

Human tRNA (uracil-5-)-methyltransferase 2 homolog A (TRMT2A) is the dedicated enzyme for the methylation of uridine 54 in transfer RNA (tRNA). Human TRMT2A has also been described as a modifier of polyglutamine (polyQ)-derived neuronal toxicity. The corresponding human polyQ pathologies include Huntington’s disease and constitute a family of devastating neuro­degenerative diseases. A polyQ tract in the corresponding disease-linked protein causes neuronal death and symptoms such as impaired motor function, as well as cognitive impairment. In polyQ disease models, silencing of TRMT2A reduced polyQ-associated cell death and polyQ protein aggregation, suggesting this protein as a valid drug target against this class of disorders. In this paper, the 1.6 Å resolution crystal structure of the RNA-recognition motif (RRM) from *Drosophila melanogaster*, which is a homolog of human TRMT2A, is described and analysed.

## Abstract 2

The 1.6 Å resolution crystal structure of the RNA-recognition motif of *D. melanogaster* tRNA (uracil-5-)-methyltransferase homolog A is reported.

## 1. Introduction

RNAs have been described to be post-transcriptionally modified with more than 170 chemical alterations installed by a multitude of enzymes or enzyme complexes. Amongst different RNA species, tRNA is the most heavily modified class, with tRNA modification and its abrogation being associated with different disease states (Orellana *et al.*, 2022 ▸; Suzuki, 2021 ▸).

Several nucleotides in the T-loop of tRNA are modified during evolution, such as m^1^A58 or m^5^U54, suggesting an important role of these modifications. In humans, tRNA methyltransferase 2 homolog A (*hs*TRMT2A) is the dedicated enzyme for the methylation of uridine at position 54 of cytosolic tRNA, whereas tRNA methyltransferase 2 homolog B (TRMT2B) methylates mitochondrial tRNA (Carter *et al.*, 2019 ▸; Powell & Minczuk, 2020 ▸). The *Escherichia coli* paralog TrmA has been associated with increased efficiency and fidelity of protein translation *in vitro* (Davanloo *et al.*, 1979 ▸; Kersten *et al.*, 1981 ▸). However, the biological function of TRMT2A and TRMT2B beyond the methylation of U54 in metazoa has largely been unexplored. A recent observation suggested *hs*TRMT2A to be involved in modulating translation fidelity (Witzenberger *et al.*, 2023 ▸).

PolyQ diseases constitute a group of diseases that are autosomally dominantly inherited and linked to an expanded cytosine–adenine–guanine (CAG) tract in the respective disease-linked gene. In patients with Huntington’s disease (HD) the disease-linked *Huntingtin* gene harbours a pathologically long CAG tract that is transcribed and then translated into an uninterrupted polyglutamine (polyQ) tract. Accumulation of the aggregation-prone Huntingtin protein, CAG repeat-containing RNA and the associated toxic gain of function interfere with normal cellular function on various levels (Bates *et al.*, 2015 ▸; Bennett *et al.*, 2007 ▸; Berendzen *et al.*, 2016 ▸). Various approaches to ameliorate disease symptoms or to slow disease progression have included attempts to reduce mutant *Huntingtin* mRNA or the mutant protein and therefore protein aggregation (Estevez-Fraga *et al.*, 2022 ▸; Tabrizi *et al.*, 2019 ▸). A promising clinical trial using antisense oligo­nucleotides to degrade mutant *Huntingtin* mRNA was recently halted (Tabrizi *et al.*, 2019 ▸; Generation HD1, NCT03761849). Therefore, the need for other strategies to lower polyQ-induced aggregation and toxicity is pressing.

A high-throughput RNAi knockdown screen using a *Drosophila melanogaster* HD disease model identified dCG3808, the homolog of *hs*TRMT2A, as a novel modifier of polyQ-induced toxicity and aggregation (Vossfeldt *et al.*, 2012 ▸). TRMT2A is predicted to consist of an N-terminal RNA-recognition motif (RRM) and a C-terminal catalytic domain folded into the Rossmann motif typical of methyltransferases (Carter *et al.*, 2019 ▸). Previously, we solved the structure of *hs*TRMT2A RRM (Margreiter *et al.*, 2022 ▸). This experimental structure, together with structural predictions of the catalytic domain, allowed us to develop *in silico* predicted inhibitors of *hs*TRMT2A and a tRNA–protein binding model (Witzenberger *et al.*, 2023 ▸). In cell culture, some of these compounds showed reduced polyQ protein aggregation in a HEK cell polyQ disease model. To further confirm the validity of the results from a fly RNAi screen for a human disease model, we solved the X-ray structure of the RRM from *Drosophila* TRMT2a (*dm*TRMT2A). The structure shows the typical fold of an RRM, with high structural similarity to *hs*TRMT2A RRM, despite low sequence similarity (32%). These findings confirm the high structural and most likely functional similarity of the proteins, but also shed light on the highly versatile yet conserved structural motif class of RNA-binding domains.

## 2. Materials and methods

### 2.1. Macromolecule production

Standard molecular-biology procedures were applied for the cloning of SUMO-His-tagged *dm*TRMT2A RRM (57–137) (Table 1 ▸). The *dm*TRMT2A RRM DNA sequence was PCR-amplified from a template using Phusion polymerase (Thermo). The correctly sized PCR product was excised for purification with a NucleoSpin Gel and PCR Clean-Up kit (Qiagen). Gibson cloning of *dm*TRMT2A RRM with pOPINS3C vector was performed using an InFusion HD cloning kit (Takara). For this purpose, pOPINS3C was linear­ized with the restriction enzymes KpnI and NcoI. Re­ligation was prevented by 5′-dephosphorylation with FastAP thermosensitive alkaline phosphatase (Thermo). Finally, 3–10 µl of InFusion reaction was transformed into *Escherichia coli* DH5α cells.

The expression of native SUMO-tagged *dm*TRMT2A RRM (57–137) in *E. coli* Rosetta (DE3) cells was induced with 0.5 *M* isopropyl β-d-1-thiogalactopyranoside at an OD of 0.6. The protein was expressed in ampicillin-supplemented LB at 37°C for 3 h. The bacterial cells were harvested by centrifugation for 20 min at 4°C at 5000*g*. For cell lysis, the pellet was resuspended in lysis buffer (500 m*M* NaCl, 50 m*M* HEPES pH 8.5, 20 m*M* imidazole, 0.5% Tween, 2% glycerol) containing one Pierce Protease Inhibitor tablet (Roche). Typically, 25 ml lysis buffer was added per 3 l of culture. The resuspended bacterial cell pellet was sonified at 4°C with a Branson sonifier 250 (Emerson) 3–4 times for 6 min at an amplitude of 40%. Insoluble cellular debris was separated from soluble *E. coli*-expressed proteins by centrifugation for 30 min at 4°C at 20 000*g*. Before proceeding with further purification steps, the supernatant was filtered with a 2.7 µm filter (Whatman). The clarified and filtered supernatant was loaded onto a 5 ml HisTrap FF column (GE Healthcare) equilibrated with His-*A* buffer (500 m*M* NaCl, 50 m*M* HEPES pH 8.5, 20 m*M* imidazole). The protein was then washed with 10 column volumes (CV) of His-*A* buffer (500 m*M* NaCl, 50 m*M* HEPES pH 8.5, 20 m*M* imidazole) followed by washing with 10 CV of high-salt-containing His-*B* buffer (2000 m*M* NaCl, 50 m*M* HEPES pH 8.5, 20 m*M* imidazole). SUMO-tagged protein was eluted with a 10 CV gradient of His-*A* buffer and His-*C* elution buffer (500 m*M* NaCl, 50 m*M* HEPES pH 8.5, 500 m*M* imidazole). For SUMO-tag cleavage, the eluate was supplemented with 100 µg PreScission protease and dialyzed against dialysis buffer (500 m*M* NaCl, 50 m*M* HEPES pH 7.5, 1 m*M* DTT) in 6.0 S tubing (ZelluTrans, Carl Roth) overnight at 4°C. The next day, the cleaved protein was applied onto an equili­brated subtractive HisTrap FF column and the protein-containing flowthrough was collected. This flowthrough was concentrated to 2 ml with a centrifugal filter (Amicon Ultra, Merck, 3K cutoff) and loaded onto a Superdex 75 (10/300 GL) size-exclusion chromatography column equilibrated in SEC buffer (500 m*M* NaCl, 50 m*M* HEPES pH 7.5). The protein-containing fractions were combined and concentrated to 8 mg ml^−1^ (Supplementary Fig. S1). Aliquots were flash-frozen in liquid nitrogen and stored at −80°C.

### 2.2. Crystallization

Crystallization experiments for the *dm*TRMT2A RRM domain were performed at the X-ray Crystallography Platform at Helmholtz Zentrum München. *dm*TRMT2A RRM crystals grew in 2 *M* ammonium sulfate, 2 *M* NaCl (Ammonium Sulfate Screen, Hampton Research) at 292 K using a protein concentration of 4–8 mg ml^−1^ (Table 2 ▸). Heavy-atom-containing compounds were soaked into *dm*TRMT2A RRM crystals to obtain a data set for phase calculation and hence structure determination of the protein by single-wavelength anomalous dispersion (SAD). For soaking, 10 m*M* stock solution was added to the drop to obtain a final concentration of 1–3 m*M*. For screening, Ta_6_Br_12_, NaBr and the derivatives Pt2 [(NH_4_)_2_PtCl_4_] and Au3 (NaAuCl_4_) from the Heavy Atom Screen (Hampton Research) were used. The soaked crystals were cooled either directly or after 24 h equilibration. 30%(*v*/*v*) ethylene glycol was used as a cryoprotectant.

### 2.3. Data collection and processing

Diffraction data were collected from crystals cooled to 100 K on the X06DA beamline at Swiss Light Source (SLS), Villigen, Switzerland. Heavy-atom-containing crystals were measured to obtain the structure of *dm*TRMT2A RRM. A fluorescence scan was performed on the mounted crystal to identify the respective heavy-atom absorption edge and thus determine the X-ray energy needed for data collection. The diffraction data were indexed and integrated using *XDS* (Kabsch, 2010 ▸) and scaled using *SCALA* (Evans, 2006 ▸). Intensities were converted to structure-factor amplitudes using *TRUNCATE* (French & Wilson, 1978 ▸). Data-collection and processing statistics are presented in Table 3 ▸. Only the data set for the crystals soaked with the heavy-atom compound NaAuCl_4_ contained strong anomalous signal that was useful for further steps.

### 2.4. Structure solution and refinement

The structure of *dm*TRMT2A RRM (57–137) was solved using the SAD protocol of *Auto-Rickshaw*, the EMBL Hamburg automated crystal structure-determination platform (Panjikar *et al.*, 2005 ▸, 2009 ▸). The input diffraction data were prepared and converted for use in *Auto-Rickshaw* using programs from the *CCP*4 suite (Agirre *et al.*, 2023 ▸). *F*_A_ values were calculated with *SHELXC* (Sheldrick, 2010 ▸). On the basis of an initial analysis of the data, the maximum resolution for substructure determination and initial phase calculation was set to 2.3 Å. Eight heavy-atom positions were located with *SHELXD* (Sheldrick, 2010 ▸). The correct hand of the sub­structure was determined with *ABS* (Hao, 2004 ▸) and *SHELXE* (Sheldrick, 2010 ▸). The occupancies of all substructure atoms were refined with *MLPHARE* from the *CCP*4 suite (Agirre *et al.*, 2023 ▸) and the phases were improved by density modification with *DM* (Cowtan & Zhang, 1999 ▸). The initial model was partially built with *ARP*/*wARP* (Morris *et al.*, 2004 ▸; Perrakis *et al.*, 2001 ▸). Further model building and refinement were performed with *Coot* (Emsley *et al.*, 2010 ▸) and *REFMAC*5 (Murshudov *et al.*, 2011 ▸), respectively, with the maximum-likelihood target function including anisotropic refinement. The final model was characterized by *R*_work_ and *R*_free_ factors of 14.1% and 19.9%, respectively (Table 4 ▸). Stereochemical analysis of the final model with *PROCHECK* (Laskowski *et al.*, 1993 ▸) showed no residues with generously allowed or unfavourable backbone dihedral angles, whereas 93% of all residues are in the core region of the Ramachandran plot. The final model was deposited in the Protein Data Bank (https://www.rcsb.org) with PDB code 7pv5.

## 3. Results and discussion

**Figure 1:**
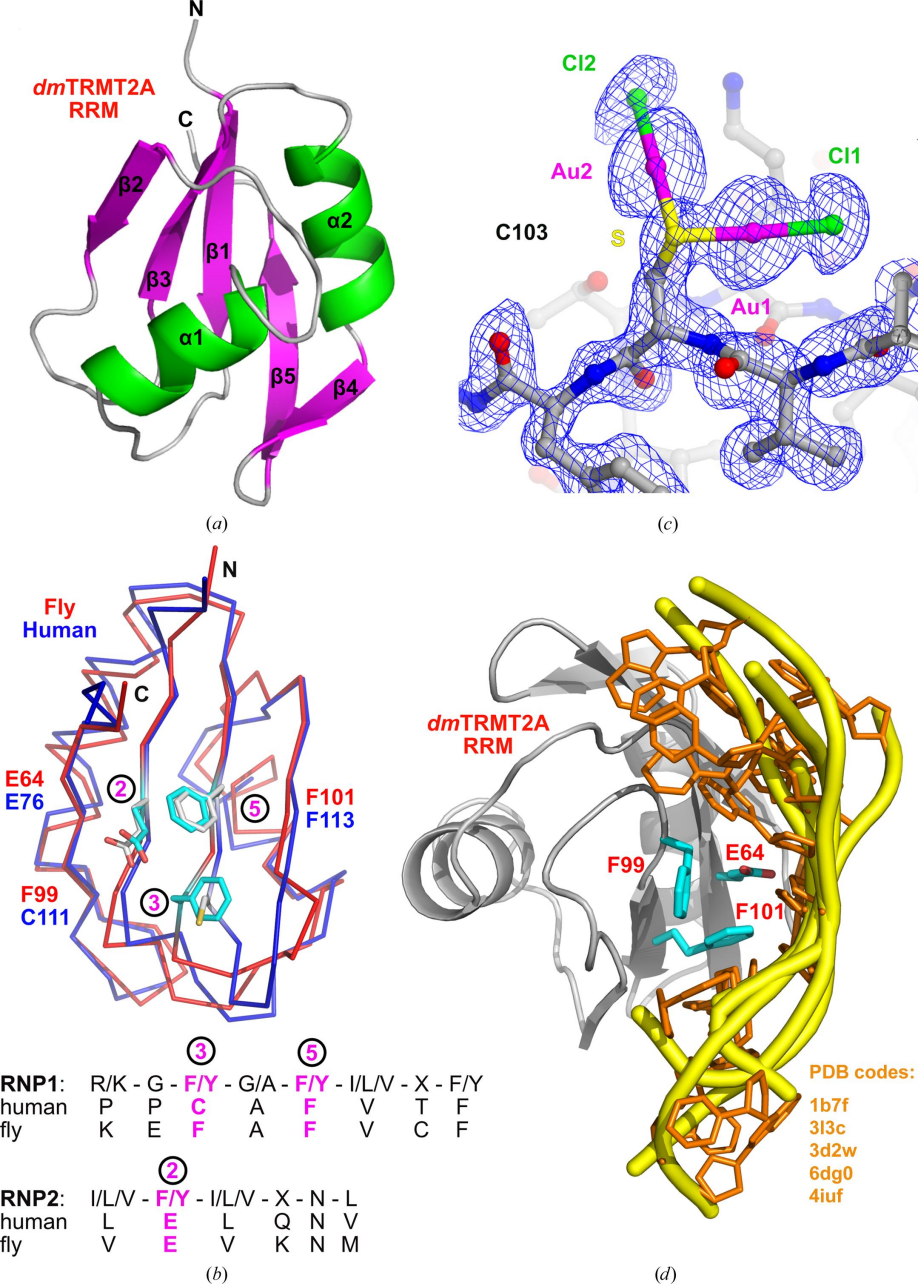
(*a*) The crystal structure of *D. melanogaster* TRMT2A RRM, fragment 57–137, at 1.6 Å resolution. The structure is shown as a ribbon coloured according to the labelled secondary-structure elements. (*b*) The superposition of *dm*TRMT2A (PDB entry 7pv5, shown as a red ribbon) and *hs*TRMT2A RRM (PDB entry 7nto, shown as a navy blue ribbon). The putative and conserved RNA-binding residues are depicted. The sequence alignment below shows the positions of the consensus RNA-binding platforms RNP1 and RNP2 in human and fly TRMT2A. Conserved RNA-binding residues at positions 3 and 5 of RNP1 and position 2 of RNP2 are indicated in magenta. (*c*) A 2*F*_o_ − *F*_c_ electron-density map contoured at 1σ is shown for the modified Cys103 residues. After soaking the crystals with NaAuCl_4_, the cysteine residue was chemically modified at its S atom with two AuCl moieties. (*d*) Superposition of the *dm*TRMT2A RRM domain (PDB entry 7pv5, shown as a grey cartoon) with its homologous RRM structures in complex with nucleic acids as identified by the *PDBeFold* search. The amino-acid residues in the *dm*TRMT2A RRM domain indicated in (*b*) are shown as blue sticks. For clarity, only nucleic acids are shown (as yellow cartoons and orange sticks) in the overlapped homologous structures. The following structures were used for the *in silico* analysis: human U1 small nuclear ribonucleoprotein A with glmS ribozyme derived from *B. anthracis* (PDB entry 3l3c; Cochrane *et al.*, 2009 ▸), the Sex-lethal (Sxl) protein of *D. melanogaster* in complex with ssRNA (PDB entry 1b7f; Handa *et al.*, 1999 ▸), the C-terminal RRM2 domain of mouse TDP-43 in complex with single-stranded DNA (PDB entry 3d2w; Kuo *et al.*, 2009 ▸), human TDP-43 RRM1–DNA complex (PDB entry 4iuf; Kuo *et al.*, 2014 ▸) and *Caenorhabditis elegans* MEC-8 C-terminal RRM domain bound to AGCACA (PDB entry 6dg0; H. Soufari & C. D. Mackereth, unpublished work).

To obtain a three-dimensional experimental model of the RNA-recognition domain (RRM) of *D. melanogaster* tRNA (uracil-5-)-methyltransferase homolog A, the protein fragment comprising amino acids 57–137 was successfully cloned, expressed, purified to homogeneity and crystallized. To solve the phase problem, the crystals were soaked with different heavy-atom derivatives. However, only one of them, NaAuCl_4_, allowed us to obtain sufficient anomalous signal. The anomalous diffraction data were collected at the Au *L*_III_ absorption edge at a wavelength of 1.0366445 Å. The structure was solved using the single-wavelength anomalous dispersion method as implemented in the *Auto-Rickshaw* pipeline (Panjikar *et al.*, 2005 ▸, 2009 ▸). The *dm*TRMT2A 57–137 fragment poses the typical domain fold of an RRM, with a five-stranded antiparallel β­-sheet and two α-helices (Fig. 1 ▸ *a*) with the topology β1–α1–β2–β3–α2–β4–β5. It highly resembles the recently published structure of the RRM from human TRMT2A (*hs*TRMT2A; Margreiter *et al.*, 2022 ▸; Fig. 1 ▸ *b*). These protein fragments share 32% sequence identity. The crystals of *dm*TRMT2A RRM contain two molecules in the asymmetric unit, with an r.m.s.d. of 0.39 Å for 74 superimposed C^α^ atoms (Supplementary Fig. S2). The NaAuCl_4_ compound used to derivatize the crystals caused chemical modification of the surface Cys103. The S atom of Cys103 has been covalently modified by two AuCl moieties (Fig. 1 ▸ *c*).

Previously, conserved consensus regions in the two middle β-sheets have been identified as a canonical RNA-binding platform where RNA nucleotides interact with solvent-exposed aromatic residues. They were termed ribonucleoprotein 1 (RNP1) and ribonucleoprotein 2 (RNP2) (Cléry & Allain, 2011 ▸; Fig. 1 ▸ *b*). For *dm*TRMT2A RRM, RNP1 consists of **Lys97**-Glu98-**Phe99**-**Ala100**-**Phe101**-**Val102**-Cys103-**Phe104** and RNP2 consists of **Val63**-Glu64-**Val65**-Lys66-**Asn67**-Met68 (where the conserved *dm*TRMT2A residues are shown in bold). Positions 3 and 5 of RNP1, as well as position 2 of RNP2 (all three of which are underlined), are the respective RNA-binding residues (Cléry & Allain, 2011 ▸; Fig. 1 ▸ *b*). The structure of *dm*TRMT2A RRM shows the expected conserved RNA-binding residues Phe99 and Phe101 in positions 3 and 5 of RNP1, whereas the nonconserved residue Glu64 is found in position 2 of RNP2. A comparison with human TRMT2A showed that Glu76 of its RRM is in the same position 2 in RNP2, as well as Cys111 and Phe113 in positions 3 and 5 in RNP1, respectively (Fig. 1 ▸ *b*). To further investigate the importance of Glu64, Phe99 and Phe101 for the interaction with nucleic acids, we performed an *in silico* analysis. Using the *PDBeFold* protein structure comparison service at the European Bioinformatics Institute (https://www.ebi.ac.uk/msd-srv/ssm; Krissinel & Henrick, 2004 ▸), we identified structurally similar proteins in complex with nucleic acids and superimposed them onto the structural model of *dm*TRMT2A RRM (Fig. 1 ▸ *d*). In the analysed co-complexes, the nucleic acids interact with the solvent-exposed parts of the β-sheet where RNP1 and RNP2 are located. Phe101 emerges as being particularly crucial, as it is fully conserved across the analysed homologous structures (Supplementary Table S1). Its involvement in nucleic acid interaction is marked by hydrophobic stacking with the bases (Fig. 1 ▸ *d*). In contrast, Glu64 and Phe99, which lack conservation, make a minor contribution (Glu64) or no contribution (Phe99) to the binding event. This emphasizes the specificity of Phe101 (Phe113 in the human protein) in mediating the interactions between the protein and nucleic acids and its central role in this molecular-recognition mechanism. While the binding mode of nucleic acids to the analysed RRM domains remains largely consistent, it is important to acknowledge the possibility of variations in the interaction in the case of the *Drosophila* or human homologs. To better understand such differences and their impact on RNA–protein binding, additional experiments will be required.

**Figure 2:**
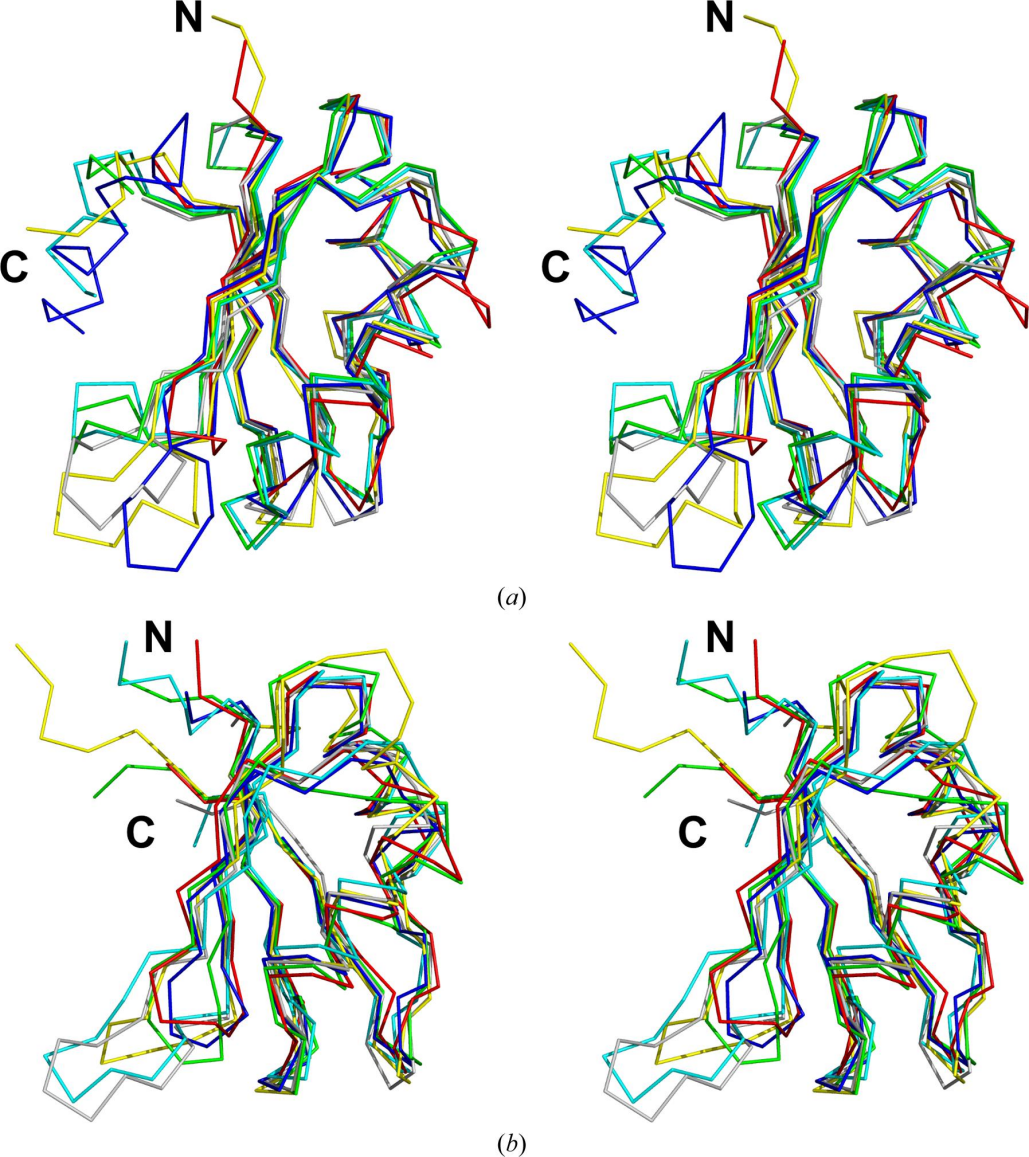
(*a*) A stereoview of the crystal structure of *dm*TRMT2A RRM superimposed with the top five results from the *PDBeFold* search. The selected structures show the lowest r.m.s.d. compared with *dm*TRMT2A RRM. The structures are shown as ribbons and coloured as follows: *dm*TRMT2A, PDB entry 7pv5, red; PDB entry 6f4j, cyan (Weber *et al.*, 2018 ▸); PDB entry 1oia, green (Nagai *et al.*, 1990 ▸); PDB entry 4a8x, yellow (Murachelli *et al.*, 2012 ▸); PDB entry 2x1f, navy blue (Pancevac *et al.*, 2010 ▸); PDB entry 1b7f, grey (Handa *et al.*, 1999 ▸). (*b*) A stereoview of the superposition of the structures identified by *PDBeFold* to have the highest sequence identity to *dm*TRMT2A RRM. The structures are shown as ribbons and coloured as follows: *dm*TRMT2A RRM, PDB entry 7pv5, red; PDB entry 7nto, green (Margreiter *et al.*, 2022 ▸); PDB entry 5iqq, cyan (Sofos *et al.*, 2016 ▸); PDB entry 1b7f, grey (Handa *et al.*, 1999 ▸); PDB entry 6e4n, navy blue (Travis *et al.*, 2019 ▸); PDB entry 2cpx, yellow (RIKEN Structural Genomics/Proteomics Initiative, unpublished work).

A comparison of the RRM from *dm*TRMT2A with other known structures using *PDBeFold* (Krissinel & Henrick, 2004 ▸) yielded high conservation of the fold among different species. Despite very low sequence identity, the *Drosophila* structure shows very high fold similarity, as measured by the r.m.s.d. to the compared models (Fig. 2 ▸, Tables 5 ▸ and 6 ▸). So far, the most similar structure with regard to sequence identity is the abovementioned *hs*TRMT2A RRM (Margreiter *et al.*, 2022 ▸; Fig. 1 ▸ *b*; Table 6 ▸). On the other hand, it is remarkable how different protein sequences can lead to nearly identical folds. The best example is the RRM of the U1 small nuclear ribonucleoprotein A from *D. melanogaster* (PDB entry 6f4j; Weber *et al.*, 2018 ▸), which shares an r.m.s.d. of 1.14 Å with *dm*TRMT2A RRM with an insignificant sequence identity of 18% (Fig. 2 ▸ *a*, Table 5 ▸).

## Supplementary Material

PDB reference: RNA-recognition motif of TRMT2A, 7pv5

Supplementary Figures and Table. DOI: 10.1107/S2053230X24000645/ek5035sup1.pdf

## Figures and Tables

**Table 1:** Macromolecule-production information

Source organism	*D. melanogaster*
DNA source	#FI05218, Gold vector containing dGC3808 (*dm*TRMT2A) from Drosophila Genomics Center
Forward primer†	*AAGTTCTGTTTCAGGGCCCG* **ACTTCGGAAATATTCAAA**
Reverse primer†	ATGGTCTAGAAAGCTTTA **ATCGGCCGAGGCCTTGGCA**
Cloning vector	pOPINS3C
Expression vector	pOPINS3C
Expression host	*E. coli* Rosetta (DE3) strain
Complete amino-acid sequence of the construct produced‡	GPTSEIFKVEVKNMGYFGIGEFKKLLRNTLKFDVTKIKAPTRKEFAFVCFRSQEDQQRALEILNGYKWKGKVLKAHVAKASAD

† The primer sequence complementary to the selected fragment of TRMT2A is shown in bold. The stop codon in the reverse primer is underlined. The 3C PreScission protease-cleavage site sequence is shown in italics.

‡ The GP residues (underlined) at the N-terminus of the produced protein fragment are artefacts left after truncation with 3C PreScission protease.

**Table 2:** Crystallization of the *dm*TRMT2A RRM domain

Method	Vapour diffusion, hanging drop
Plate type	VDX 24-well plate
Temperature (K)	292
Protein concentration (mg ml^−1^)	4–8
Buffer composition of protein solution	500 m*M* NaCl, 50 m*M* HEPES pH 7.5
Composition of reservoir solution	2 *M* ammonium sulfate, 2 *M* NaCl
Volume and ratio of drop	3 µl, 1:1 ratio
Volume of reservoir (ml)	1

**Table 3:** Data-collection and processing statistics for *dm*TRMT2A RRM Values in parentheses are for the outer shell.

Diffraction source	Beamline X06DA, SLS
Wavelength (Å)	1.0366445
Temperature (K)	100
Detector	PILATUS 2M-F
Crystal-to-detector distance (mm)	185.057
Rotation range per image (°)	0.1
Total rotation range (°)	360
Exposure time per image (s)	0.1
Space group	*C*2
*a*, *b*, *c* (Å)	92.41, 41.89, 48.82
α, β, γ (°)	90, 110.80, 90
Mosaicity (°)	0.3
Resolution range (Å)	50–1.6 (1.64–1.60)
Total No. of reflections	150144 (9039)
No. of unique reflections	23209 (1684)
Completeness (%)	99.7 (99.0)
CC_1/2_	99.8 (91.1)
Multiplicity	6.5 (5.6)
〈*I*/σ(*I*)〉	11.6 (3.5)
*R*_r.i.m._ (%)	10.1 (43.3)
Overall *B* factor from Wilson plot (Å^2^)	11.9

**Table 4:** Structure solution and refinement Values in parentheses are for the outer shell.

Resolution range (Å)	45.64–1.60 (1.64–1.60)
Completeness (%)	99.9
σ Cutoff	*F* > 0.000σ(*F*)
No. of reflections, working set	22079 (1584)
No. of reflections, test set	1130 (99)
Final *R*_cryst_	0.141 (0.173)
Final *R*_free_	0.199 (0.323)
Cruickshank DPI	0.082
No. of non-H atoms	
Protein	1337
Solvent	147
Ions	68
Total	1552
R.m.s. deviations	
Bond lengths (Å)	0.016
Angles (°)	1.936
Average *B* factors (Å^2^)	
Protein	21.1
Solvent	40.0
Ions	38.9
Ramachandran plot	
Most favoured (%)	93
Allowed (%)	7

**Table 5:** The results of the structural similarity search performed with *PDBeFold* The structures are ordered according to their r.m.s.d. to *dm*TRMT2A RRM. The values in the table include the r.m.s.d. in Å, the number of aligned C^α^ atoms (*N*_align_) and the sequence identity in % (%seq).

R.m.s.d.	*N*_align_	%seq	PDB code/chain	Organism	Protein	Reference
1.14	65	18	6f4j/*D*	*D. melanogaster*	U1 small nuclear ribonucleoprotein A	Weber *et al.* (2018 ▸)
1.23	65	18	1oia/*B*	*H. sapiens*	U1 small nuclear ribonucleoprotein A	Nagai *et al.* (1990 ▸)
1.24	69	17	4a8x/*A*	*H. sapiens*	RNA-binding protein with serine-rich domain 1	Murachelli *et al.* (2012 ▸)
1.24	69	26	2x1f/*A*	*S. cerevisiae*	mRNA 3′-end-processing protein RNA15	Pancevac *et al.* (2010 ▸)
1.27	68	29	1b7f/*A*	*D. melanogaster*	Sex-lethal (Sxl) protein	Handa *et al.* (1999 ▸)

**Table 6:** The results of the structural similarity search performed with *PDBeFold* The structures are ordered according to their sequence identity to the *dm*TRMT2A RRM fragment. The values in the table include the r.m.s.d. in Å, the number of aligned C^α^ atoms (*N*_align_) and the sequence identity in % (%seq).

R.m.s.d.	*N*_align_	%seq	PDB code/chain	Organism	Protein	Reference
1.65	74	32	7nto/*A*	*H. sapiens*	RNA-recognition motif of TRMT2A	Margreiter *et al.* (2022 ▸)
1.65	71	30	5iqq/*E*	*H. sapiens*	RNA-recognition motif of RNA-binding protein 7	Sofos *et al.* (2016 ▸)
1.27	68	29	1b7f/*A*	*D. melanogaster*	Sex-lethal (Sxl) protein	Handa *et al.* (1999 ▸)
1.46	67	28	6e4n/*A*	*T. brucei*	RNA-recognition motif of the putative tbrgg2 protein	Travis *et al.* (2019 ▸)
1.61	68	28	2cpx/*A*	*H. sapiens*	RNA-binding domain of hypothetical protein FLJ11016	RIKEN Structural Genomics/Proteomics Initiative (unpublished work)
