# Supplementary material for: Crystal structure of the RNA-recognition motif of Drosophila melanogaster tRNA (uracil-5-)-methyltransferase homolog A
Source: Acta Crystallogr F Struct Biol Commun. 2024 Jan 25;80(Pt 2):36–42. doi: 10.1107/S2053230X24000645 (PMC10836426; doi:10.1107/S2053230X24000645)
Supplement: Supplementary file 1 [file f-80-00036-sup1.pdf]

STRUCTURAL BIOLOGY COMMUNICATIONS

Volume 80 (2024)

Supporting information for article:

Crystal structure of the RNA-recognition motif of *Drosophila melanogaster* tRNA (uracil-5-)-methyltransferase homolog A (TRMT2A)

Dierk, Monika Witzenbergen, Robert Janowski and Dierk Niessing

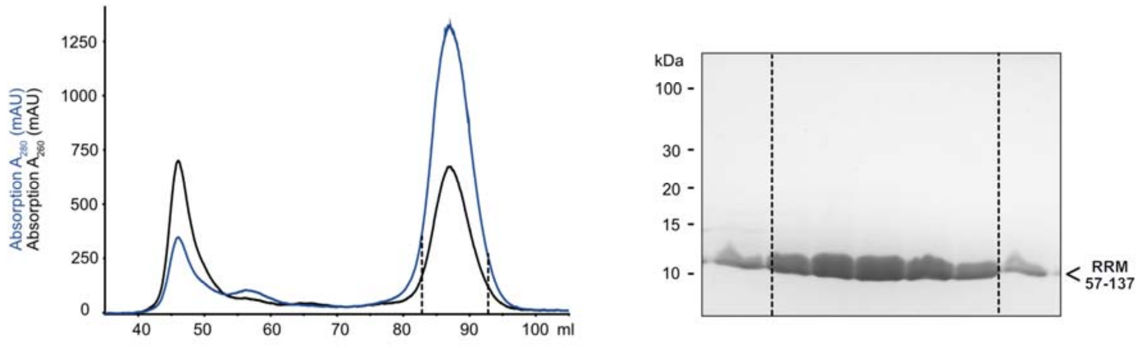

Figure S1 Elution profile of size-exclusion chromatography using a Superdex 75, (S75, 10/300 GL) column shows the last step of *dm*TRMT2A RRM (57-137) purification. A corresponding SDS PAGE (right) from fractions between both dotted lines in the elution profile (left) shows the expected size of the RRM at around 10-11 kDa (calculated molecular weight: 9.8 kDa). Dashed lines on the gel image (right) show fractions used for concentration and subsequent crystallization.

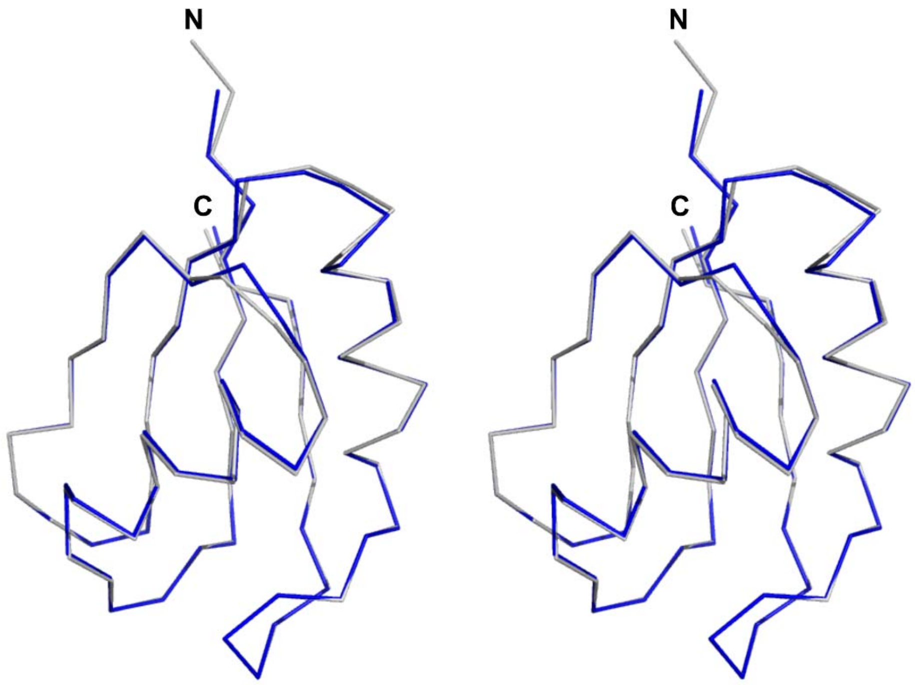

Figure S2 The crystals of *dm*TRMT2A RRM contain two molecules in the asymmetric unit with r.m.s.d value of 0.39 Å for 74 superimposed Cα atoms. The figure shows a stereo view of the overlap of both independent chains: A – navy blue and B – grey. The figure was prepared with *PyMOL* (Schrödinger and DeLano, 2020).

Table S1 Conservation of the amino acid residues in position 2 of the RNP2 and in position 3 and 5 of the RNP1 in the analysed RRM structures.

The values in the table include r.m.s.d. in Å and sequence identity in % (%seq).

PDB ID	Organism	RNP2 Position 2	RNP1 Position 3	RNP1 Position 5	r.m.s.d.	%seq	Reference
7PV5	*D. melanogaster*	E64	F99	**F101**	-	-	This study
7NTO	*H. sapiens*	E76	C111	**F113**	1.65	32	Margreiter *et al.*, 2022
1B7F	*D. melanogaster*	I128	Y168	**F170**	1.27	29	Handa *et al.*, 1999
3L3C	*H. sapiens*	Y13	Q54	**F56**	1.33	18	Cochrane *et al.*, 2009
3D2W	*M. musculus*	F194	F229	**F231**	1.79	22	Kuo *et al.*, 2009
4IUF	*H. sapiens*	L107	F147	**F149**	1.51	17	Kuo *et al.*, 2014
6DG0	*C. elegans*	F24	V60	**F62**	1.43	16	unpublished
